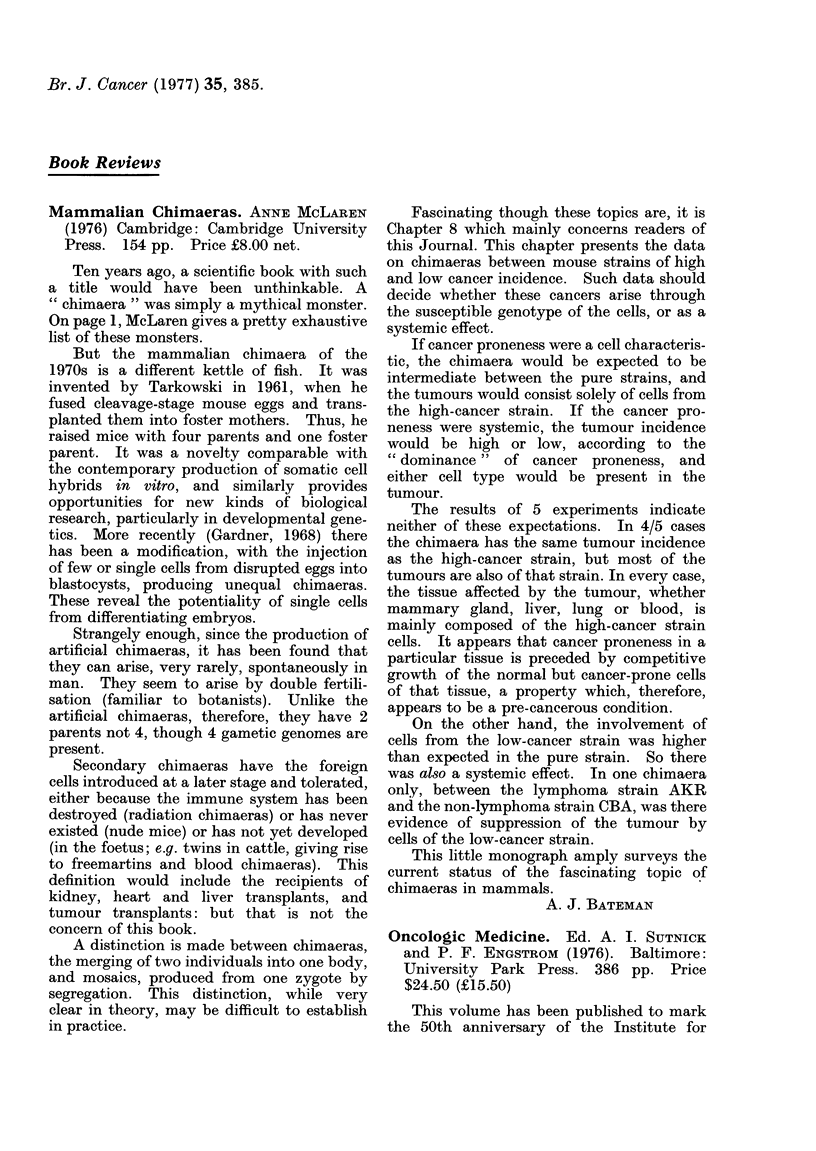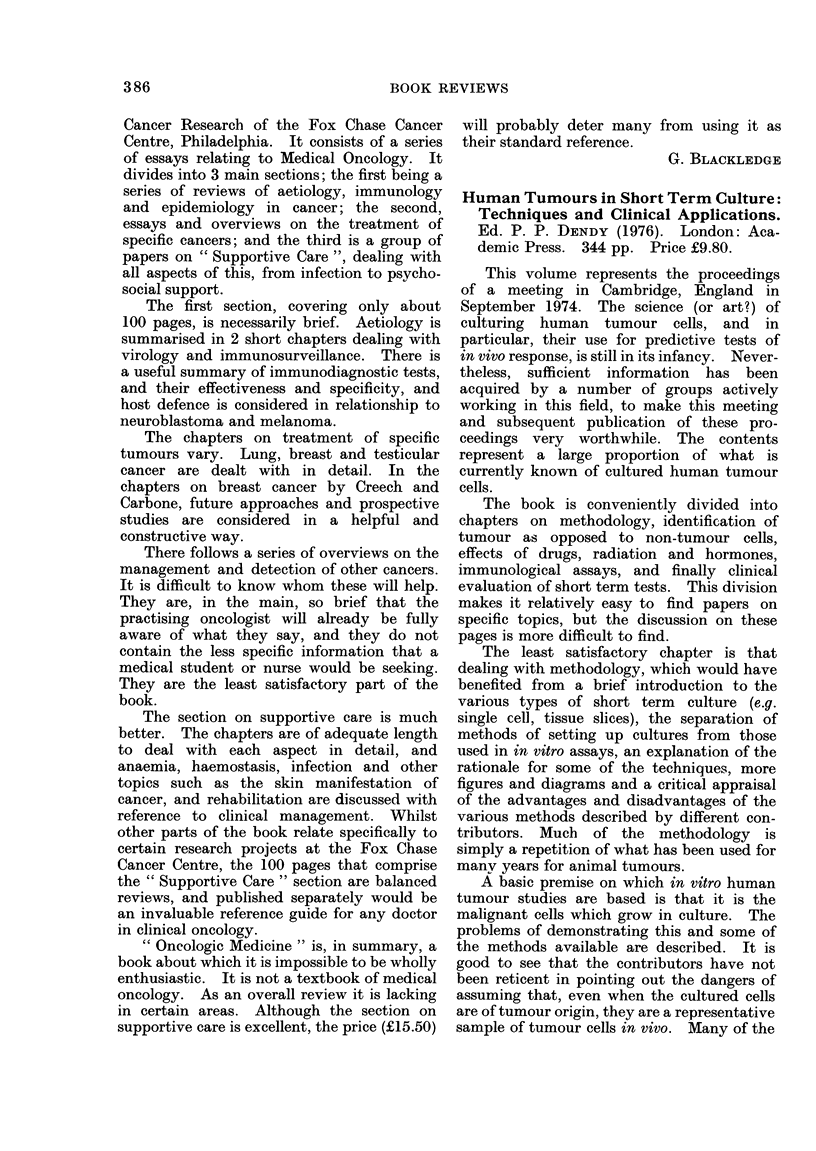# Oncologic Medicine

**Published:** 1977-03

**Authors:** G. Blackledge


**Oncologic Medicine.** Ed. A. I. Sutnick and P. F. Engstrom (1976). Baltimore: University Park Press. 386 pp. Price $24.50 (£15.50)

This volume has been published to mark the 50th anniversary of the Institute for

Cancer Research of the Fox Chase Cancer Centre, Philadelphia. It consists of a series of essays relating to Medical Oncology. It divides into 3 main sections; the first being a series of reviews of aetiology, immunology and epidemiology in cancer; the second, essays and overviews on the treatment of specific cancers; and the third is a group of papers on " Supportive Care ", dealing with all aspects of this, from infection to psychosocial support.

The first section, covering only about 100 pages, is necessarily brief. Aetiology is summarised in 2 short chapters dealing with virology and immunosurveillance. There is a useful summary of immunodiagnostic tests, and their effectiveness and specificity, and host defence is considered in relationship to neuroblastoma and melanoma.

The chapters on treatment of specific tumours vary. Lung, breast and testicular cancer are dealt with in detail. In the chapters on breast cancer by Creech and Carbone, future approaches and prospective studies are considered in a helpful and constructive way.

There follows a series of overviews on the management and detection of other cancers. It is difficult to know whom these will help. They are, in the main, so brief that the practising oncologist will already be fully aware of what they say, and they do not contain the less specific information that a medical student or nurse would be seeking. They are the least satisfactory part of the book.

The section on supportive care is much better. The chapters are of adequate length to deal with each aspect in detail, and anaemia, haemostasis, infection and other topics such as the skin manifestation of cancer, and rehabilitation are discussed with reference to clinical management. Whilst other parts of the book relate specifically to certain research projects at the Fox Chase Cancer Centre, the 100 pages that comprise the " Supportive Care " section are balanced reviews, and published separately would be an invaluable reference guide for any doctor in clinical oncology.

" Oncologic Medicine " is, in summary, a book about which it is impossible to be wholly enthusiastic. It is not a textbook of medical oncology. As an overall review it is lacking in certain areas. Although the section on supportive care is excellent, the price (£15.50) will probably deter many from using it as their standard reference.

G. Blackledge